# Soil applied silicon and manganese combined with foliar application of 5-aminolevulinic acid mediate photosynthetic recovery in Cd-stressed *Salvia miltiorrhiza* by regulating Cd-transporter genes

**DOI:** 10.3389/fpls.2022.1011872

**Published:** 2022-09-29

**Authors:** Yuee Sun, Xin Li, Ullah Najeeb, Zhuoni Hou, Noman Ali Buttar, Zongqi Yang, Basharat Ali, Ling Xu

**Affiliations:** ^1^ Zhejiang Province Key Laboratory of Plant Secondary Metabolism and Regulation, College of Life Sciences and Medicine, Zhejiang Sci-Tech University, Hangzhou, China; ^2^ Faculty of Science, Universiti Brunei Darussalam, Jalan Tungku Link, Gadong, Brunei; ^3^ Department of Agricultural Engineering, Khwaja Fareed University of Engineering and Information Technology (KFUEIT), Rahim Yar Khan, Pakistan

**Keywords:** heavy metals, plant growth regulator, silicon, manganese, oxidative stress, gene regulation

## Abstract

*Salvia miltiorrhiza* is an important medicinal plant that experiences significant growth and biomass losses when cultivated on cadmium (Cd) contaminated soils. High Cd accumulation in plant tissues also increases the risk of metal entry into the food chain. In this study, we proposed that Cd accumulation in *S. miltiorrhiza* can be restricted through plant growth regulators and nutrient management. Therefore, *S. miltiorrhiza* seedlings were transplanted into mixed nutrient soil for two weeks, then treated with 30 mg kg^-1^ CdCl_2_, 200 mg kg^-1^ Na_2_SiO_3_·9H_2_O, and 100 mg kg^-1^ MnSO_4_, and simultaneously sprayed with 10 mg L^-1^ ALA on the leaves one week later. This study showed that elevated Cd accumulation significantly reduced plant growth and biomass. This growth inhibition damaged photosynthetic machinery and impaired carbon assimilation. In contrast, 5-aminolevulinic acid (ALA) significantly promoted the biomass of *S. miltiorrhiza*, and the dry weight of plants treated with ALA combined with manganese (Mn)/silicon (Si) increased by 42% and 55% as compared with Cd+Mn and Cd+Si treatments. Exogenously applied ALA and Si/Mn significantly activated antioxidant enzymes and promoted the growth recovery of *S. miltiorrhiza*. Further, exogenous ALA also reduced the Cd concentration in *S. miltiorrhiza*, especially when combined with Si. Compared with the Cd+Si treatment, the Cd+Si+ALA treatment reduced the Cd concentration in roots and leaves by 59% and 60%, respectively. Gene expression analysis suggested that ALA and Si significantly up-regulated genes associated with Cd transport. Other genes related to heavy metal tolerance mechanisms are also regulated to cope with heavy metal stress. These results indicated that the combined action of ALA and Si/Mn could reduce Cd-toxicity by increasing chlorophyll content and changing oxidative stress and can also affect Cd accumulation by regulating gene expression.

## Introduction

With rapid urbanization, and industrial and anthropogenic activities (such as smelting and mining), agricultural soils and waters in many developing countries are seriously contaminated with heavy metals. For instance, human activities such as mining, pesticides, fertilizers, and industrial wastewater have significantly increased cadmium (Cd) concentration in soils (Nicholson et al., 2003). The national soil contamination survey report showed that the total point exceedance rate of the national soil was 16%, and Cd occupies the largest part, 7%. Cd contamination is rapidly increasing throughout the country, with more than 50% in the southwest region and 10%-40% in northern, northeastern, and western China (MEP and MLR, 2014). In China, about 19.4% of arable land is contaminated with heavy metals (Zhao et al., 2015), including 2.8×10^5^ hm^2^ of farmland contaminated with Cd (Liu et al., 2015). Cadmium, nickel, lead and arsenic were considered the most harmful pollutants in soil (Shang et al., 2018; Mwamba et al., 2020; Aslam et al., 2021). Cadmium is a non-essential element for plant growth. In soils, it is quickly mobilized, absorbed by plant roots, and accumulated in edible parts, endangering human health (Ali et al., 2014; Hussain et al., 2020). It can also inhibit the uptake and transport of some nutrients, causing nutrient deficiencies and slow growth in plants (Mwamba et al., 2016; Chattha et al., 2021). Plants cultivated on these contaminated soils experience significant growth and developmental challenges. The excess reactive oxygen species (ROS) in plant tissues can damage plant growth (Tian et al., 2014; Yang et al., 2018). Heavy metal contamination accelerates the process of ROS production, disrupting cell structure and affecting cell function (Daud et al., 2009; Najeeb et al., 2011).

In Cd-contaminated soils, plant growth can be managed through appropriate nutrient management and/or plant growth regulator applications. For instance, manganese (Mn) is an essential trace element for plant growth (Li et al., 2019). It can reduce Cd absorption by forming Cd-Mn complexes in root tissues (Srivastava and Dubey, 2011; Wang et al., 2021a). However, the concentration of Mn should not be too high as it can limit the uptake of other nutrients, such as calcium, magnesium, iron, or zinc (Loren et al., 2021). Similarly, silicon (Si) is the second most abundant mineral element in soil, comprising approximately 28% of the earth’s crust; it can interact with heavy metals, reducing their bioavailability and absorption by plants (Malčovská et al., 2014; Adrees et al., 2015). In Cd-stressed plants, Si accumulates in the cell wall, forming a Cd complex (Si-wall matrix), reducing Cd absorption (Ma et al., 2015; Xu et al., 2017; Thind et al., 2020). Toxic metals in soil or growth media can slow root growth and development, which can be restored by Si treatment (Zhang et al., 2008a; Lu et al., 2018; Tian et al., 2020). Exogenous Si application has been found effective in reducing Cd absorption and protecting cellular organelles from Cd injury by regulating metal transporter genes (Gheshlaghpour et al., 2021). 5-aminolevulinic acid (ALA), a key precursor of pyrrole molecule synthesis pathways such as chlorophyll (Akram and Ashraf, 2013; Gill et al., 2015; Gill et al., 2016), can promote plant performance under stressed environments (Ali et al., 2013b; Xu et al., 2016; Xu et al., 2018). It also activates the antioxidant enzyme defence system, reduces lipid peroxidation, and protects plant organelles from damage (Youssef and Awad, 2008; Xu et al., 2015). Further, El-Shora et al. (2021) also found that ALA reduced malondialdehyde (MDA) and ROS levels in *S. miltiorrhiza* and improved plant growth under lead stress. ALA was also effective in protecting chlorophyll machinery and the photosynthesis process of plants under Cd-stressed environments (Ali et al., 2013a).

*Salvia miltiorrhiza* L. mainly contains fat-soluble and water-soluble practical components (Chen et al., 2021; Yu et al., 2021; Chen et al., 2022b), and its roots are commonly used in the pharmaceutical industry to treat cardiovascular and cerebrovascular diseases (Xing et al., 2015; Zhang et al., 2021; Chen et al., 2022a). The accumulation of Cd can seriously affect its efficacy and quality, affecting human health. Therefore, it is necessary to limit the Cd content in plant tissues to maintain the stability of the efficacy of *S. miltiorrhiza*. However, most studies are conducted on food crops, and few have been conducted on reducing Cd content in the medicinal plant *S. miltiorrhiza*. In this study, Cd accumulation in *S. miltiorrhiza* could be suppressed through soil management (Mn and Si) and plant growth regulator ALA application. To date, synergistic effects of ALA combined with MnSO_4_/Na_2_SiO_3_ on *S. miltiorrhiza* under heavy metal Cd stress have not been studied. This study explored the effects of ALA and Si/Mn on Cd-stressed *S. miltiorrhiza*, intending to understand the physiological pathways associated with Cd absorption and transport in plant tissues.

## Materials and methods

### Plant material

*S. miltiorrhiza* seeds were collected from Shangluo, Shanxi Province, China. Soak the prepared seeds in distilled water for 12 hours. Then, the seeds were placed in a seedling tray and grown at 28°C for seven days, constantly replenishing water during the cultivation process. All the plants were transplanted into long flowerpots and grown under 200 μmol m^−2^ s^−1^ active photon flux density, 24/20°C (day/night temperature), 60-70% relative humidity, and 14/10 hours (light/night) photoperiod. After two months of growth, seedlings were transplanted into pots (180 cm) containing nutrient soil, vermiculite, and perlite (4: 2: 1, v:v) as substrate. The *S. miltiorrhiza* seedlings were transplanted into mixed nutrient soil for two weeks, then treated with 30 mg kg^-1^ CdCl_2_, 200 mg kg^-1^ Na_2_SiO_3_·9H_2_O, and 100 mg kg^-1^ MnSO_4_, and sprayed with 10 mg L^-1^ ALA on the leaves one week later. Among them, the concentrations of CdCl_2_, Na_2_SiO_3_·9H_2_O, MnSO_4,_ and ALA were selected based on preliminary experiments where several Cd levels, i.e., 0, 10, 30, and 50 mg kg^-1^ were tested. The results showed that Cd concentrations at 10 mg kg^-1^ did not significantly affect plant growth, whereas Cd at 50 mg kg^-1^ was more toxic to plant growth, causing the leaves to be yellowed and even die. There were ten different treatments, namely control, Cd, Si, Mn, ALA, Cd+Si, Cd+Mn, Cd+ALA, Cd+Si+ALA, and Cd+Mn+ALA. Three independent replicates of each treatment were used, and three plants per replicate were selected for subsequent analysis. Seven days after the treatment, the plants were harvested for biomass measurements and biochemical analysis.

### Chlorophyll and carotenoid content

To estimate chlorophyll contents, fresh root and leaf samples (0.1 g) were placed in a centrifugal tube containing 4.5 mL absolute ethanol, 4.5 mL acetone, and 1 mL distilled water and incubated in the dark overnight. The absorbance of the solution was taken at 663 nm, 645 nm, and 470 nm (Porra et al., 1989).

### Malondialdehyde and reactive oxygen species

The MDA content was measured by mixing 5 mL of 0.5% 2-thiobarbituric acid with 1.5 mL of enzyme solution, and then it was bathed in water at 95°C for 30 min, followed by a rapid ice bath and centrifugation. The supernatant was measured at 532 nm and 600 nm (Zhou and Leul, 1999). Superoxide radicles $\left({\text{O}}_{2}^{-}\right)$ were measured as described by Jiang and Zhang (2001). Add 0.1g of fresh roots or leaves to 3 mL of pre-cooled potassium phosphate buffer solution, grind, and centrifuge. The absorbance was measured at 530 nm to calculate the $\left({\text{O}}_{2}^{-}\right)$ generation rate. The content of hydrogen peroxide (H_2_O_2_) was determined by mixing 0.1g fresh sample with 2 mL of 0.1% trichloroacetic acid (TCA) according to the method of Velikova et al. (2000). Hydroxyl ion (^-^OH) contents were measured following Halliwell et al. (1987). Fresh samples (0.1 g) were ground in 1 mL sodium phosphate buffer solution and centrifuged. Hydroxyl ion contents of the supernatant solution were measured at 550 nm.

### Antioxidant enzyme activities

Fresh samples (0.1 g) were ground with potassium phosphate buffer solution and centrifuged, and the supernatant solution was used for further analysis. Superoxide dismutase (SOD) activity was determined by the photochemical nitro blue tetrazolium (NBT) method of Zhang et al. (2008b), and the absorbance was measured at 560 nm. Peroxidase (POD) activity was determined by the method of Zhou and Leul (1999). The change of absorbance of the reaction system at 470 nm was measured, and finally, POD activity was calculated. Catalase (CAT) was measured at 240 nm, and the activity of CAT was calculated Aebi (1984). Ascorbic peroxidase (APX) was measured by Nakano and Asada (1981), and the absorbance change within 1 min was measured at 290 nm. The absorbance at 340 nm to calculate glutathione reductase activity (GR) by Jiang and Zhang (2002).

### Tissue cadmium contents

Dried *S. miltiorrhiza* root and leaf samples (0.05 g) were digested with 10 mL of nitric acid in a polytetrafluoroethylene container into a microwave digestion apparatus. The digested samples were poured into a Teflon beaker, heated to nearly 1 mL, and adjusted the volume to 50 mL in a centrifuge tube. The iCAP™ RQ ICP-MS (Thermo Fisher, USA) was used to determine Cd content in the roots and leaves of *S. miltiorrhiza*. The bioaccumulation quantity (BCQ) was calculated as the product of Cd concentration in different tissues and their dry weights.

### Scanning electron microscopy

Two modifiers of MnSO_4_ and Na_2_SiO_3_·9H_2_O were scattered on two conductive tapes, respectively, then blew off the weak samples with a rubber suction bulb and stuck the conductive tapes on the aluminum sample table. A scanning electron microscope (ZEISS GeminiSEM 300, Germany) was used to characterize the two kinds of particles, and their microstructure was observed.

### Gene expression analysis

RNA from fresh roots and leaves of *S. miltiorrhiza* was extracted using a TaKaRa MiniBEST Plant RNA Extraction Kit (Takara Bio, Kyoto, Japan). The extracted RNA was quantitatively analyzed through agarose gel electrophoresis and Nanodrop 2000 spectrophotometer (Hu et al., 2021). Then, the cDNA was synthesized using TaKaRa PrimeScript™ RT Master Mix (Perfect for Real-Time). Three repeated experiments were conducted for the next step of data analysis. TB Green™ Premix Ex Taq™II (Tli RNaseH Plus) (TaKaRa) was applied to a real-time PCR reaction. *Actin*, a constant expression internal reference gene, was selected as a control. The quantitative experiment was carried out with QuantStudio 6 Flex Real-Time PCR Systems. The primers for RT-PCR reaction were designed by GenScript and shown in **Supplementary Table 1**. Among them, primers *PAL*, *C4H*, *DXS2*, *DXR*, *HMGR3*, and *Actin* were from Xing et al. (2018), primers *CSD2*, *FSD2*, *MSD1*, *MSD2* were from Han et al. (2020), primer *PPT* was from Liu et al. (2019), primer *ERF73* was from Zheng et al. (2021). The relative expression level was expressed by the calculation result of the 2^-ΔΔCt^ values, according to Han et al. (2022).

### Tanshinone and salvianolic acid

The weighed dried roots and leaves (0.02 g) were placed in a centrifuge tube containing 1 mL of 70% methanol. Place the prepared sample overnight, and then use an ultrasonic cleaning machine for ultrasonic treatment. The centrifuged supernatant was collected with a disposable syringe and filtered with a 0.22 μm filter membrane to obtain the required sample. Three technical repetitions were performed for each treatment. The reagent concentrations in plant tissues were determined by Waters e2695 HPLC and Waters 2998 UV detector. The HPLC method was consistent with that of Liu et al. (2016). Cryptotanshinone, tanshinone I, and tanshinone IIA contents were determined at 270 nm, and salvianolic acid B was determined at 288 nm.

### Statistical analysis

SPSS v23.0 (SPSS, Chicago, IL, USA) was used to compare the mean value of three replicates ± standard error (SE) for data analysis. The statistical chart was drawn by GraphPad Prism 9. One-way ANOVA was performed, then LSD and Duncan tests were used, and the significance level P< 0.05 was selected for data processing.

## Results

### ALA and Mn/Si promote the growth of *S. miltiorrhiza*

**Figure 1:**
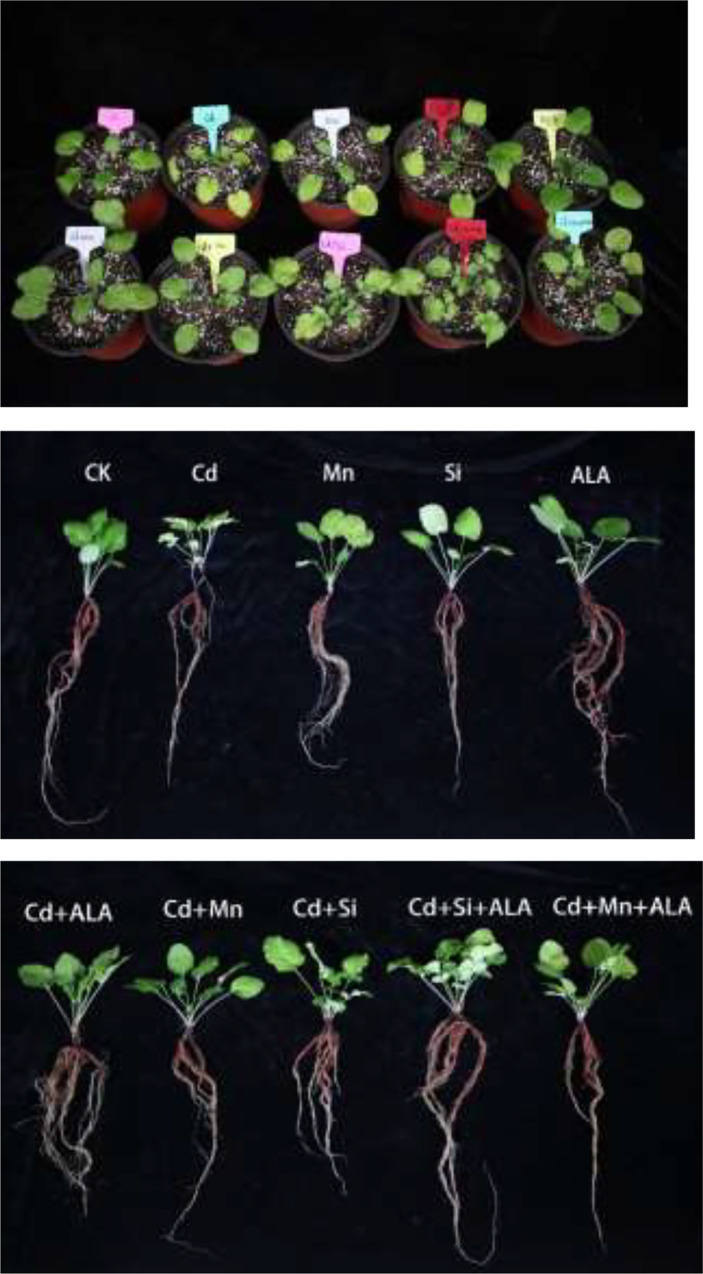
Effects of application of 5-aminolevulinic acid (ALA), silicon (Si), manganese (Mn), ALA combined with Mn/Si on *S. miltiorrhiza* plants growth under cadmium (Cd) stress. Concentrations of Cd, ALA, Si and Mn were 30 mg kg^-1^ CdCl_2_, 10 mg L^-1^ ALA, 200 mg kg^-1^ Na_2_SiO_3_·9H_2_O and 100 mg kg^-1^ MnSO_4_, respectively.

Cadmium treatment significantly reduced plant growth and biomass production in *S. miltiorrhiza* (**Figure 1**). Compared with non-stressed control, Cd-stressed plants produced 36% and 42% lower root and leaf dry weights, respectively (**Table 1**). ALA significantly promoted the growth of *S. miltiorrhiza* plants in Cd- and non-stressed environments. Under Cd-free treatment, Mn-treated plants produced 28% and 36% less root and leaf biomass, and Si-treated plants produced 22% and 40% less. ALA-treated plants had 82% and 56% more root and leaf biomass, respectively, compared with the control (**Table 1**). However, adding ALA, Si and Mn significantly reduced the inhibitory effect of Cd on plant growth and altered these growth parameters. ALA increased root, and leaf dry weight of Cd stressed plants by 36% and 39%, Mn increased the dry weight of roots and leaves by 27% and 36%, and Si increased the dry weight of roots and leaves by 45% and 72%, respectively, compared with Cd only treated plants. However, the combination treatment of ALA and Si increased the dry weight of roots and leaves by 1.24 and 1.19 folds, and the combination treatment of ALA and Mn increased by 0.8 and 1.04 folds, respectively, compared with Cd alone. Therefore, the combined ALA and Si/Mn treatment alleviated plant growth under Cd stress. Compared with the control, the Cd-treated plant contained nearly half of the leaf chlorophyll a, chlorophyll b, and carotenoid contents (**Table 2**). Compared with Cd alone, the chlorophyll content of plants increased significantly after adding ALA, Mn, and Si; among them, the total chlorophyll content increased by 29%, 19%, 26%, and the carotenoid content increased by 3%, 11%, 24%, respectively. However, treatments of Cd+Si+ALA and Cd+Mn+ALA significantly increased the total chlorophyll content by 42% and 64% compared with Cd+Si, and Cd+Mn stress alone (**Table 2**). Therefore, spraying of ALA in Cd+Si and Cd+Mn showed a better effect on increasing chlorophyll levels.

**Table 1:** Effects of ALA and Mn/Si application on the biomass (g) of root and leaf of Cd stressed plants.

Treatments	Root (g)		Leaf (g)	
	FW	DW	FW	DW
CK	2.31 ± 0.12d	0.30 ± 0.02cd	1.74 ± 0.07b	0.22 ± 0.02c
ALA	4.23 ± 0.12a	0.54 ± 0.03a	2.37 ± 0.22a	0.35 ± 0.03a
Cd	1.58 ± 0.13f	0.19 ± 0.03g	0.97 ± 0.05e	0.13 ± 0.02f
Mn	1.79 ± 0.09ef	0.21 ± 0.01fg	1.08 ± 0.09e	0.14 ± 0.02ef
Si	1.72 ± 0.09f	0.23 ± 0.01efg	1.01 ± 0.08e	0.13 ± 0.02f
Cd+ALA	2.18 ± 0.12d	0.26 ± 0.04def	1.46 ± 0.05c	0.18 ± 0.01d
Cd+Mn	1.97 ± 0.12e	0.24 ± 0.03ef	1.28 ± 0.10d	0.18 ± 0.01de
Cd+Si	2.25 ± 0.08d	0.27 ± 0.02de	1.40 ± 0.07cd	0.22 ± 0.02c
Cd+Si+ALA	3.20 ± 0.16b	0.42 ± 0.03b	1.71 ± 0.05b	0.29 ± 0.01b
Cd+Mn+ALA	2.75 ± 0.13c	0.34 ± 0.01c	1.70 ± 0.07b	0.27 ± 0.01b

Concentrations of Cd, ALA, Si and Mn were 30 mg kg^-1^ CdCl_2_, 10 mg L^-1^ ALA, 200 mg kg^-1^ Na_2_SiO_3_·9H_2_O and 100 mg kg^-1^ MnSO_4_, respectively. Data are the means of three replicates (mean ± SE). Small letters indicate significant differences at P< 0.05 by Duncan’s multiple range tests. DW, Dry weight; FW, Fresh weight.

**Table 2:** Effects of ALA and Mn/Si application on chlorophyll a, chlorophyll b, total chlorophyll, and carotenoid of Cd stressed plants.

Treatments	Chlorophyll a (mg g^−1^ FW)	Chlorophyll b (mg g^−1^ FW)	Total chlorophyll (mg g^−1^ FW)	Carotenoid (mg g^−1^ FW)
CK	0.37 ± 0.020ab	0.13 ± 0.009ab	0.50 ± 0.028ab	0.09 ± 0.021a
ALA	0.40 ± 0.028a	0.14 ± 0.009a	0.54 ± 0.034a	0.08 ± 0.013a
Cd	0.19 ± 0.002d	0.07 ± 0.004d	0.26 ± 0.003d	0.04 ± 0.001b
Cd+ALA	0.23 ± 0.005c	0.10 ± 0.015c	0.33 ± 0.020c	0.05 ± 0.011b
Cd+Mn	0.22 ± 0.006c	0.08 ± 0.006cd	0.30 ± 0.001cd	0.05 ± 0.005b
Cd+Si	0.23 ± 0.024c	0.09 ± 0.006c	0.32 ± 0.031c	0.06 ± 0.003b
Cd+Si+ALA	0.34 ± 0.040b	0.12 ± 0.013b	0.46 ± 0.052b	0.10 ± 0.026a
Cd+Mn+ALA	0.35 ± 0.006b	0.15 ± 0.015a	0.50 ± 0.009ab	0.09 ± 0.006a

Concentrations of Cd, ALA, Si and Mn were 30 mg kg^-1^ CdCl_2_, 10 mg L^-1^ ALA, 200 mg kg^-1^ Na_2_SiO_3_·9H_2_O and 100 mg kg^-1^ MnSO_4_, respectively. Data are the means of three replicates (mean ± SE). Small letters indicate significant differences at P< 0.05 by Duncan’s multiple range tests. FW, Fresh weight.

### Reactive oxygen species and MDA contents under the application of ALA and Mn/Si

**Figure 2:**
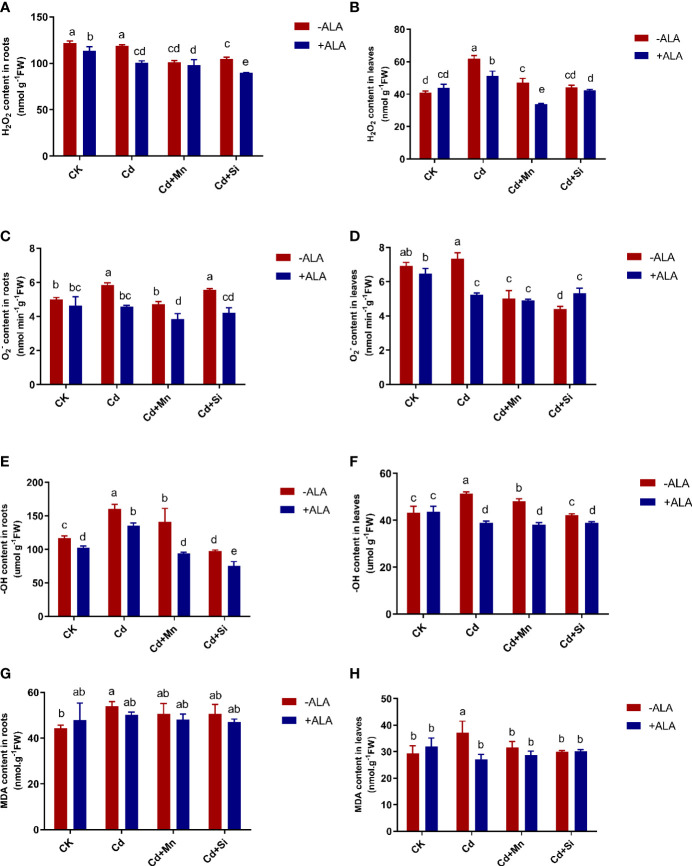
Effects of application of ALA and Mn/Si on levels of reactive oxygen species and lipid peroxidation in the roots and leaves of Cd stress plants. Hydrogen peroxide (H_2_O_2_) in the roots **(A)** and leaves **(B)**; superoxide radical $\left({\text{O}}_{2}^{-}\right)$ in the roots **(C)** and leaves **(D)**; hydroxyl ion (^-^OH) content in the roots **(E)** and leaves **(F)**; malondialdehyde (MDA) in the roots **(G)** and leaves **(H)**. Concentrations of Cd, ALA, Si and Mn were 30 mg kg^-1^ CdCl_2_, 10 mg L^-1^ ALA, 200 mg kg^-1^ Na_2_SiO_3_·9H_2_O and 100 mg kg^-1^ MnSO_4_, respectively. Data are the means of three replicates (mean ± SE). Small letters indicate significant differences at P< 0.05 by Duncan’s multiple range tests.

In this study, Cd treatment alone significantly increased ROS levels in the roots and leaves of *S. miltiorrhiza* compared with the control. In this case, the combined application of ALA and Mn/Si achieved the maximum reduction of ROS accumulation, which alleviated the adverse effects of excessive ROS on *S. miltiorrhiza* plants. For example, the H_2_O_2_ content in leaves increased by 51% under Cd treatment compared with untreated plants. After ALA and Mn/Si treatments, H_2_O_2_ in roots and leaves was significantly reduced compared to Cd stress (**Figures 2A, B**). In terms of changes in $\left({\text{O}}_{2}^{-}\right)$ content, in root tissue, ALA and Mn alone or in combination significantly reduced $\left({\text{O}}_{2}^{-}\right)$ under Cd treatment, while Si had no significant effect on it; for leaves, both ALA and Mn/Si alone or in combination significantly reduced $\left({\text{O}}_{2}^{-}\right)$ content. For example, under ALA, Mn, Si, ALA+Si, and ALA+Mn treatments, the $\left({\text{O}}_{2}^{-}\right)$ content in roots was reduced by 22%, 19%, 5%, 30%, and 34%, and the $\left({\text{O}}_{2}^{-}\right)$ content of leaves was reduced by 29%, 32%, 40%, 27%, and 33%, respectively, compared with the Cd treatment (**Figures 2C, D**).

Similarly, ALA, Mn, Si, ALA+Si, and ALA+Mn reduced the ^-^OH content of roots by 16%, 12%, 40%, 53%, and 42%, and the ^-^OH content of leaves by 24%, 6%, 18%, 24%, and 26%, respectively, compared with the Cd treatment. The combined ALA and Mn/Si treatments were more significant than the single treatment. For example, when ALA was applied together with Mn or Si, the ^-^OH content of roots was reduced by 22% and 34%, respectively, compared with the Mn and Si treatment alone (**Figures 2E, F**). Cd stress significantly increased MDA content in both root and leaf tissues; however, this accumulation was reversed by ALA and Mn/Si treatments (**Figures 2G, H**). Further, ALA and Mn/Si application also had a significant synergistic effect on reducing ROS levels in *S. miltiorrhiza* under Cd stress.

### Antioxidant enzyme activities contents under the application of ALA and Mn/Si

**Figure 3:**
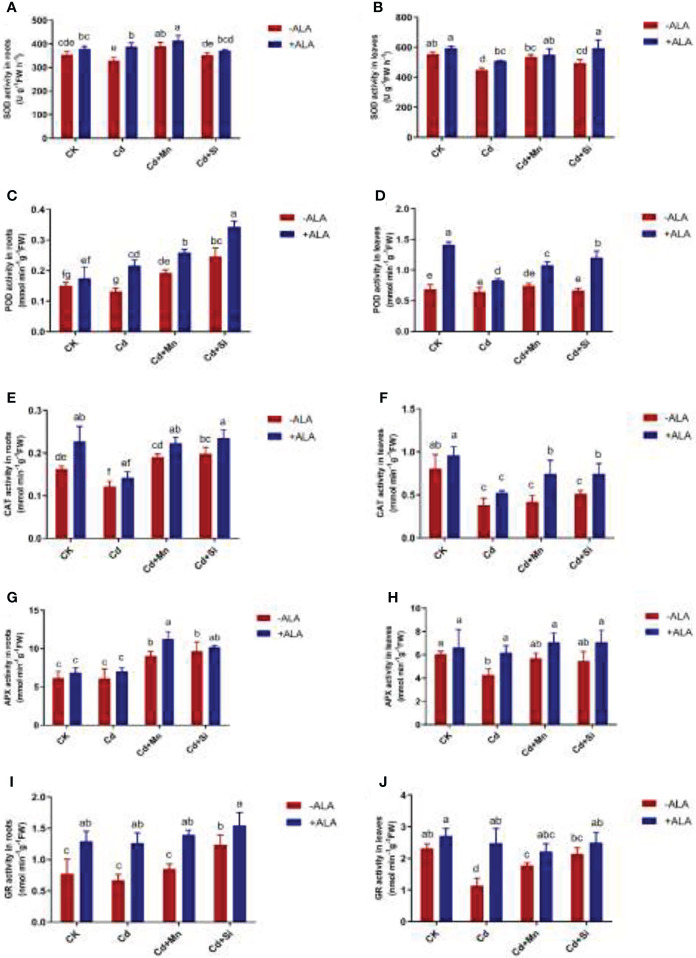
Effects of application of ALA and Mn/Si on antioxidant enzyme activities in the roots and leaves of Cd stress plants. Superoxide dismutase (SOD) in the roots **(A)** and leaves **(B)**; peroxidase (POD) in the roots **(C)** and leaves **(D)**; catalase (CAT) in the roots **(E)** and leaves **(F)**; ascorbic acid peroxidase (APX) in the roots **(G)** and leaves **(H)**; glutathione reductase (GR) in the roots **(I)** and leaves **(J)**. Concentrations of Cd, ALA, Si and Mn were 30 mg kg^-1^ CdCl_2_, 10 mg L^-1^ ALA, 200 mg kg^-1^ Na_2_SiO_3_·9H_2_O and 100 mg kg^-1^ MnSO_4_, respectively. Data are the means of three replicates (mean ± SE). Small letters indicate significant differences at P< 0.05 by Duncan’s multiple range tests.

Antioxidant enzyme activities such as SOD, POD, CAT, APX, and GR in roots and leaves of *S. miltiorrhiza* were significantly reduced when plants responded to Cd stress. However, exogenous ALA and Si/Mn application significantly improved the activity of antioxidant enzymes in root and leaf tissues, particularly when applied in combination (**Figure 3**). The SOD activity was highest under Cd+Si+ALA treatment, which is 32% higher than that of plants treated with Cd. Compared with Cd treatment, POD also increased significantly under ALA and Si/Mn treatments, among which the activity of Cd+Si+ALA treatment in roots increased the most, with a 1.59-fold increase compared to Cd treatment. When ALA treated plants together with Cd, Cd+Mn, and Cd+Si, it was found that CAT activity gradually increased. APX showed the highest activity in the roots and leaves under Cd+Si+ALA treatment. The activity of GR was significantly increased in the plants treated with ALA. According to the above data, the antioxidant enzyme activity of plants treated with ALA and Mn/Si was higher than that in ALA-, Mn-, and Si- treated plants under Cd stress.

### Cadmium accumulation under the application of ALA and Mn/Si

**Figure 4:**
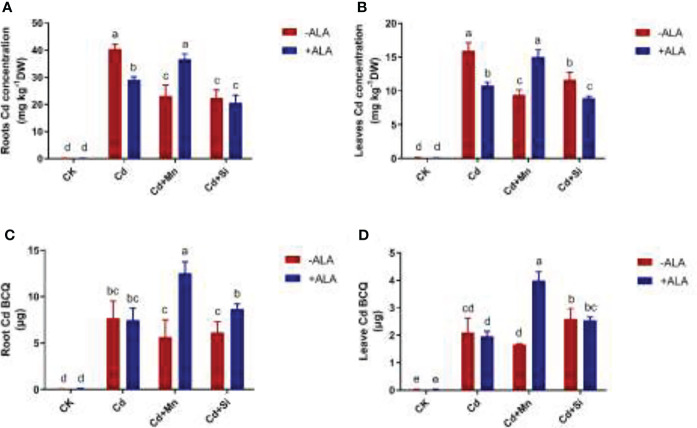
Effects of application of ALA and Mn/Si on Cd accumulation under Cd stress plants. Cd accumulation in the roots **(A)**, Cd accumulation in the leaves **(B)**, Cd bioaccumulation quantity (BCQ) in the roots **(C)**, Cd bioaccumulation quantity in the leaves **(D)**. Concentrations of Cd, ALA, Si and Mn were 30 mg kg^-1^ CdCl_2_, 10 mg L^-1^ ALA, 200 mg kg^-1^ Na_2_SiO_3_·9H_2_O and 100 mg kg^-1^ MnSO_4_, respectively. Data are the means of three replicates (mean ± SE). Small letters indicate significant differences at P< 0.05 by Duncan’s multiple range tests.

Under the application of ALA, Mn, and Si, the total BCQ of Cd in *S. miltiorrhiza* tissue decreased by 3%, 25%, and 10%, respectively, compared with Cd stress alone. Instead, spraying ALA after adding Mn or Si to Cd-contaminated soil increased the BCQ of Cd in *S. miltiorrhiza* tissue (**Figures 4C, D**). For example, Si+ALA and Mn+ALA application to Cd-stressed plants increased the total BCQ of Cd by 15% and 69%, respectively, compared with the Cd treatment alone. Among them, the BCQ in the roots increased by 13% and 63%, and in the leaves by 21% and 90%, respectively. In addition, plants subjected to Cd stress accumulated more Cd in roots and leaves compared to the control. In the roots and leaves of Cd-stressed plants, applying ALA and Si/Mn significantly reduced the Cd content (**Figures 4A, B**). The application of Cd+Si+ALA significantly reduced Cd content in roots and leaves more than in other combinations. Therefore, ALA and Si combined treatment significantly lowered Cd content.

### Gene expression level and liquid phase analysis under the application of ALA and Mn/Si

**Figure 5:**
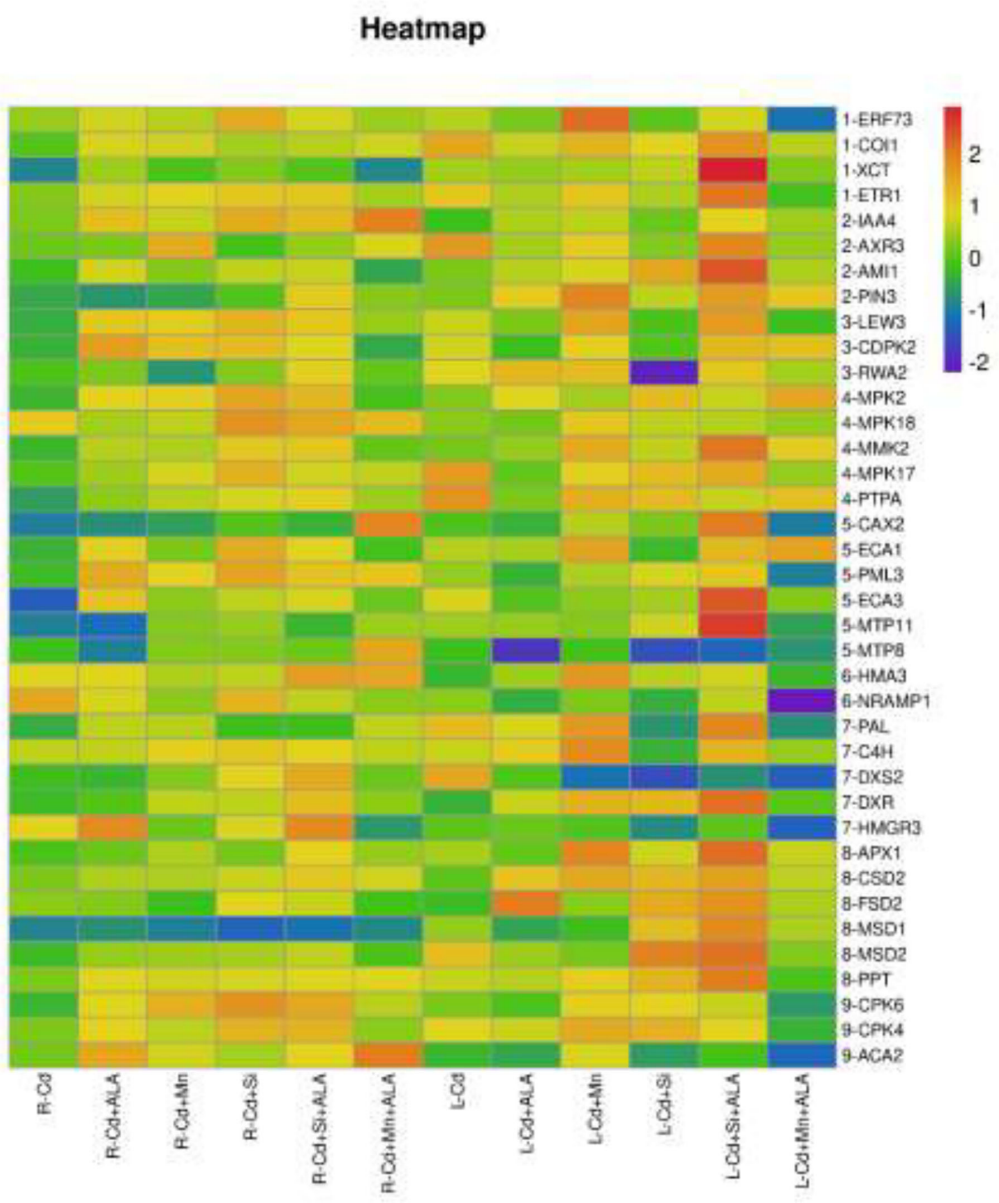
Expression maps of nine resistance-related genes of *S. miltiorrhiza*. Concentrations of Cd, ALA, Si and Mn were 30 mg kg^-1^ CdCl_2_, 10 mg L^-1^ ALA, 200 mg kg^-1^ Na_2_SiO_3_·9H_2_O and 100 mg kg^-1^ MnSO_4_, respectively. Data are the means of three replicates (mean ± SE). The expression level of the control group was normalized as “1”. Drawing heat map using expression data processed by log10.

**Figure 6:**
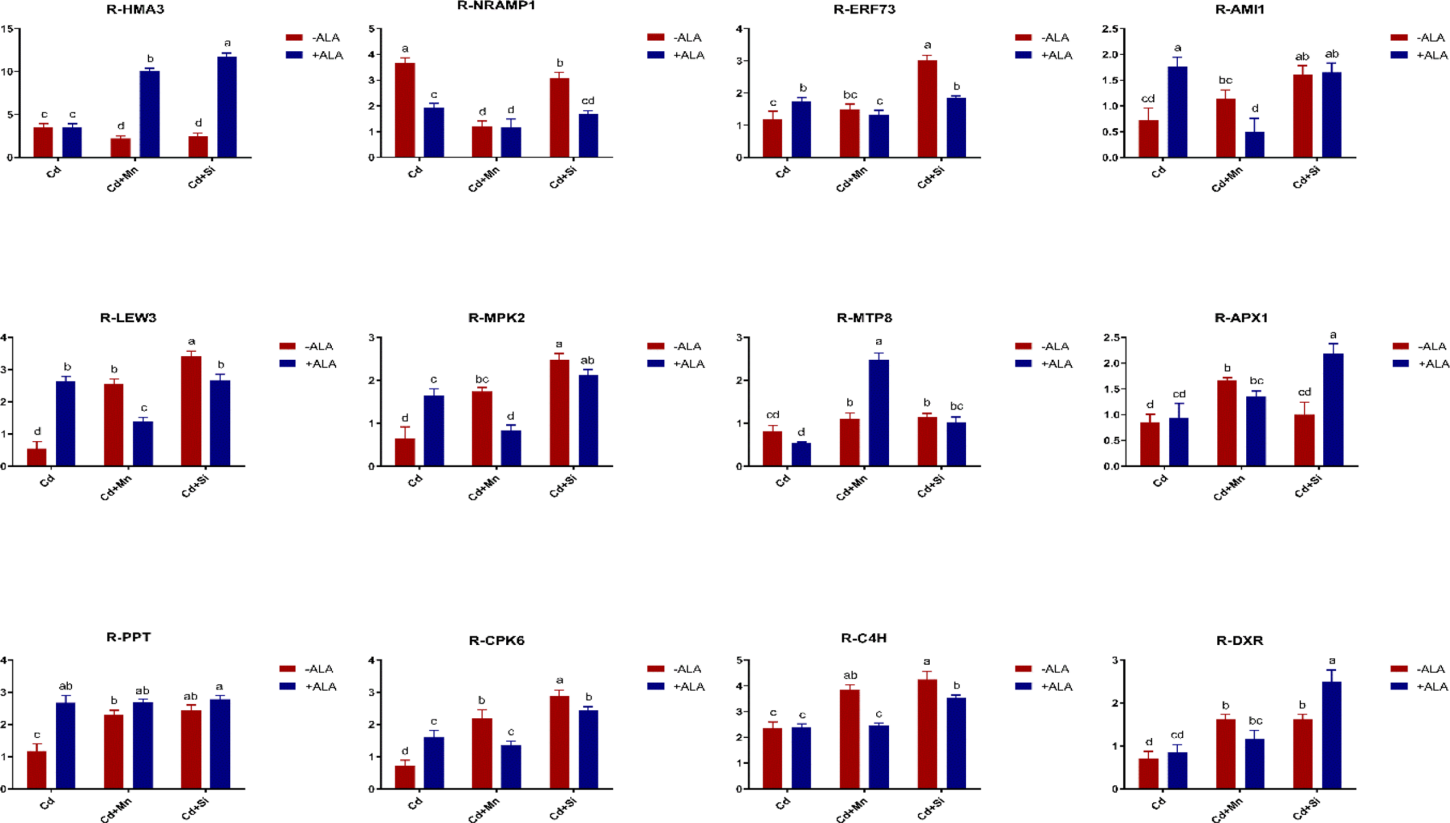
Effects of ALA and Mn/Si application on the expressions of tolerant related genes in *S. miltiorrhiza* roots under Cd stress. Concentrations of Cd, ALA, Si and Mn were 30 mg kg^-1^ CdCl_2_, 10 mg L^-1^ ALA, 200 mg kg^-1^ Na_2_SiO_3_·9H_2_O and 100 mg kg^-1^ MnSO_4_, respectively. Data are the means of three replicates (mean ± SE). Small letters indicate significant differences at P< 0.05 by Duncan’s multiple range tests.

**Figure 7:**
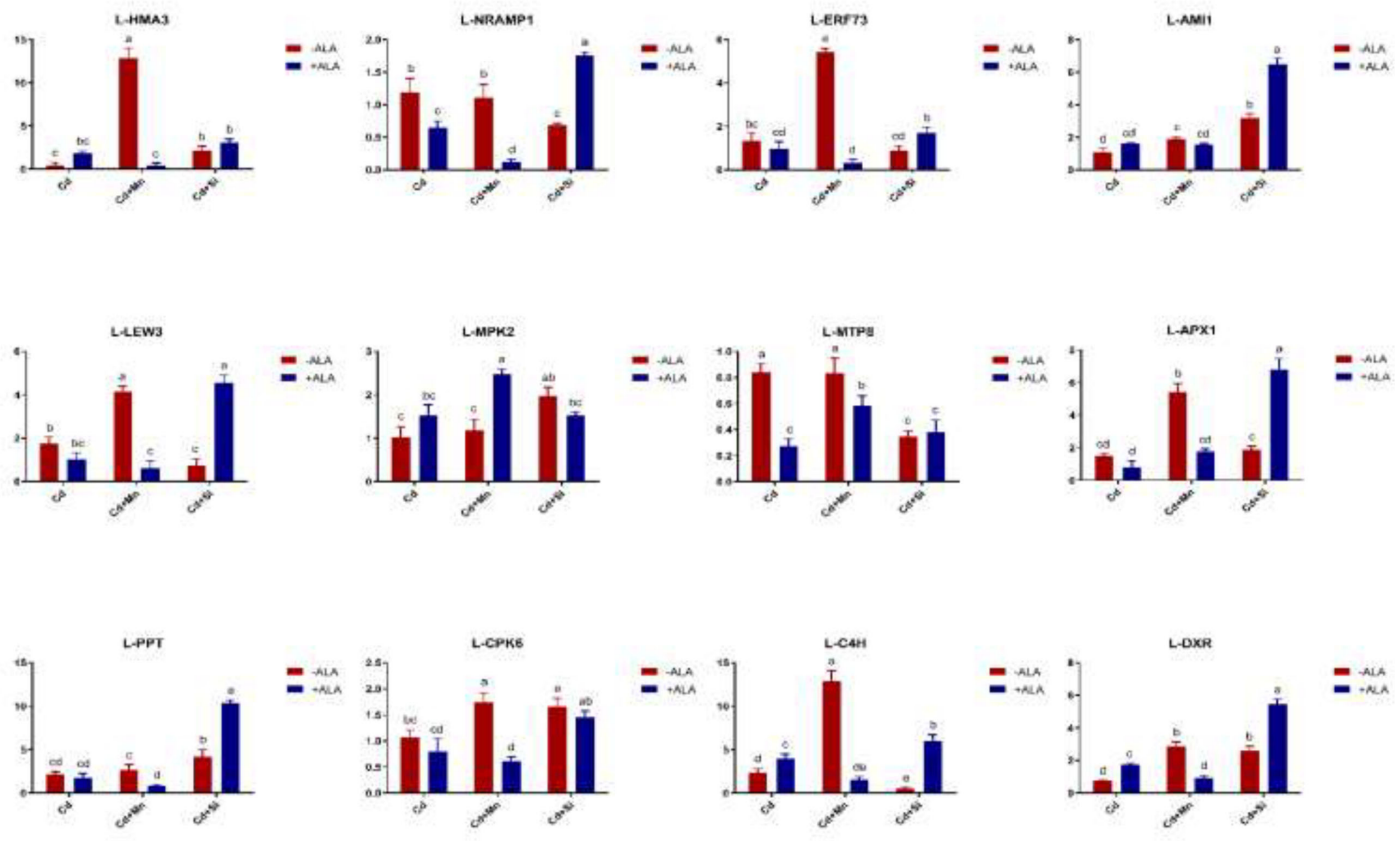
Effects of ALA and Mn/Si application on the expressions of tolerant related genes in *S. miltiorrhiza* leaves under Cd stress. Concentrations of Cd, ALA, Si and Mn were 30 mg kg^-1^ CdCl_2_, 10 mg L^-1^ ALA, 200 mg kg^-1^ Na_2_SiO_3_·9H_2_O and 100 mg kg^-1^ MnSO_4_, respectively. Data are the means of three replicates (mean ± SE). Small letters indicate significant differences at P< 0.05 by Duncan’s multiple range tests.

**Figure 8:**
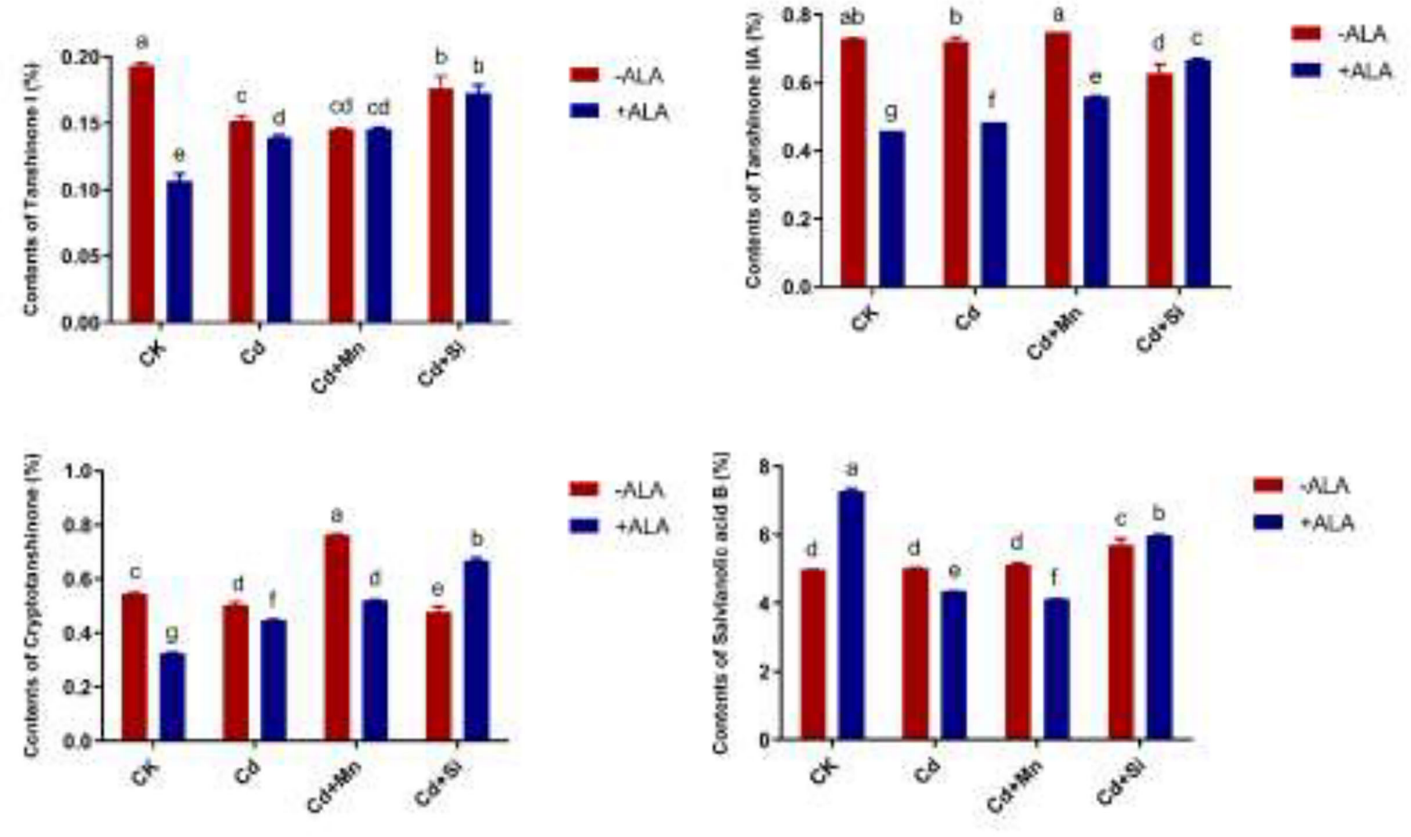
Effects of ALA and Mn/Si application on cryptotanshinone, tanshinone I, tanshinone IIA and salvianolic acid B under Cd stress plants. Concentrations of Cd, ALA, Si and Mn were 30 mg kg^-1^ CdCl_2_, 10 mg L^-1^ ALA, 200 mg kg^-1^ Na_2_SiO_3_·9H_2_O and 100 mg kg^-1^ MnSO_4_, respectively. Data are the means of three replicates (mean ± SE). Small letters indicate significant differences at P< 0.05 by Duncan’s multiple range tests.

In this study, we studied the expression levels of nine different genes, including Cd transport-related genes (*HMA3*, *NRAMP1*), Mn transport-related genes (*CAX2, ECA1, ECA3, PML3, MTP8, MTP11*), ROS scavenged genes (*APX1*, *CSD2*, *FSD2*, *MSD1*, *MSD2*, *PPT*), auxin-related genes (*IAA4*, *AXR3*, *AMI1*, *PIN3*), ABA signalling pathway genes (*LEW3*, *CDPK2*, *RWA2*), ethylene-related genes (*COI1*, *XCT*, *ETR1*, *ERF73*), calcium channel genes (*CPK4*, *CPK6*, *ACA2*), MAPK cascade reaction genes (*MPK2*, *MPK17*, *MPK18*, *MMK2*, *PTPA*) and *S. miltiorrhiza* component related genes (*PAL*, *C4H*, *DXS2*, *DXR*, *HMGR3*) (**Figure 5** and **Supplementary Table 2**). The relative expression of genes related to stress resistance in the root and leaf of *S. miltiorrhiza* under several stress conditions has been shown in **Figures 6**, **7**. In the leaves of Cd-exposed plants, application of ALA, Mn, Si, and Si+ALA significantly improved the transcript level of *HMA3* by 2.03, 1.95, 2.79, 4.03 folds, respectively, while reduced by 0.05 fold by Mn+ALA treatment. In the roots of Cd-treated plants, application of ALA, Mn, and Si significantly decreased the transcript level of *HMA3*, while the addition of Si+ALA treatments increased the transcription level of *HMA*3. In addition, the transcription level of *NRAMP1* under several treatments in roots was lower than that under cadmium alone, and the transcription level in leaves was higher only under Cd+Si+ALA treatment than under cadmium alone. In addition, ALA reduced the relative expression of genes related to *S. miltiorrhiza*, i.e., *DXS2* and *C4H* in Cd-treated plants, while Mn, Si, Si+ALA, Mn+ALA increased their expression levels. The addition of ALA, Mn, Si, Si+ALA, and Mn+ALA all significantly increased the relative expression of *PAL* and *DXR* in Cd-treated plants. Compared with the control, Cd stress reduced the contents of cryptotanshinone, tanshinone I, and tanshinone IIA by 8%, 22%, and 1%, respectively, while the contents of salvianolic acid B increased by 0.7% (**Figure 8**). Compared with Cd treatment, spraying of ALA reduced these contents, and applying Mn increased these contents. Compared with control, Si+ALA treated plants accumulated 33%, 13%, and 19% more cryptotanshinone, tanshinone I, and salvianolic acid B contents, respectively, but tanshinone IIA accumulation was reduced by 8% (**Figure 8**).

### Scanning electron microscope observation

**Figure 9:**
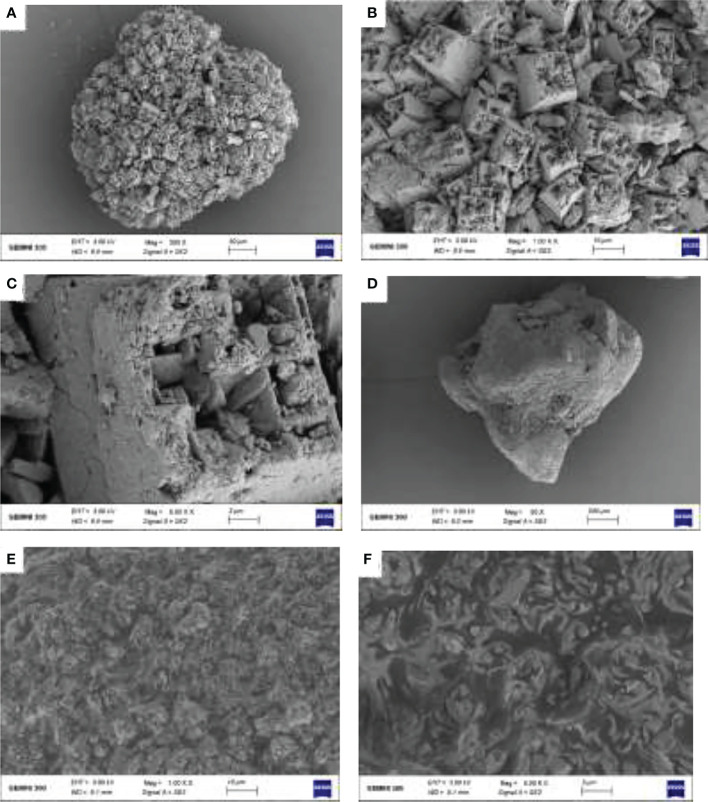
Scanning electron microscope (SEM) results of MnSO_4_ image at 30 μm **(A)**, MnSO_4_ image at 10 μm **(B)**, MnSO_4_ image at 2 μm **(C)**, Na_2_SiO_3_·9H_2_O image at 200 μm **(D)**, Na_2_SiO_3_·9H_2_O image at 10 μm **(E)**, Na_2_SiO_3_·9H_2_O image at 2 μm **(F)**.

According to SEM results, MnSO_4_ particles were larger, and a complete particle could be observed at 30 μm. Its surface pictures were taken at a voltage of 3 kV, and a working distance of 8.9 mm (**Figure 9**). The diameter of Na_2_SiO_3_·9H_2_O particles was relatively small, and a complete Na_2_SiO_3_·9H_2_O particle could be observed at 200 μm; these pictures were taken at a voltage of 3 kV and a working distance of 8.1 mm. Na_2_SiO_3_·9H_2_O has a larger particle size and surface area, making it easier for heavy metals to be fixed in polluted soil. It also improves soil pH, thus reducing the solubility of metals. However, the surface of MnSO_4_ is relatively smooth, which is quite different from that of Na_2_SiO_3_·9H_2_O, which may differ from their action mechanism. MnSO_4_ mainly reduces Cd content in plants by competing with Cd for shared ion channels.

## Discussion

In this study, the growth of *S. miltiorrhiza* under Cd stress was obviously depressed. The spraying of ALA restored the growth and increased the plant biomass (**Figure 1**). These findings are consistent with the results of Xu et al. (2021). They found that ALA could significantly increase the biomass of sunflowers under Cd stress. Similarly, Ali et al. (2013a) also reported the positive role of ALA in improving the antioxidant defence ability of *Brassica napus* under Cd toxicity. The addition of silicon made the combination of Cd and cell wall easier, limiting the extra plastic transport of Cd and reducing Cd toxicity to the cytoplasm (Ye et al., 2011). Moreover, it has been stated that Si can improve the pH value of soil, change the metal forms in soil and deposit toxic metals (Adrees et al., 2015). SEM analysis shows that Na_2_SiO_3_·9H_2_O has a large surface area, and there are many microporous structures on the surface, which can chelate with heavy metals to fix heavy metal Cd, which may have a similar effect to that of mussel shells fixing nickel (Jia et al., 2017). The new emerging plant growth regulator, ALA, can reduce ROS accumulation, lipid peroxidation, and toxicity caused by heavy metals by stimulating the activity of heme antioxidant enzymes (Youssef and Awad, 2008). Spraying ALA on leaves can promote plants’ metabolism and restore plants’ growth under abiotic stress (Akram et al., 2012). ALA application to Cd-stressed rape plants could reduce cadmium toxicity by increasing chlorophyll content, photosynthesis and biomass accumulation (Ali et al., 2013a).

The present findings demonstrated that under Cd stress, the growth of *S. miltiorrhiza* was inhibited, and biomass decreased. However, the combination of ALA and Si/Mn amended the harm of Cd stress, promoted plant growth, and increased plant biomass. The appropriate amount of ROS can improve the growth of plants under adverse conditions. In contrast, a large amount of ROS affects the metabolism of plants and causes different degrees of cell damage (Ali et al., 2013b). Cadmium pollution caused the accumulation of ROS and oxidative damage to cells and affected the growth of plants (**Figure 2**), which is consistent with the findings of Gheshlaghpour et al. (2021). The harm of ROS to plants has been confirmed in many studies (Foyer and Noctor, 2005; Farooq et al., 2013; Adrees et al., 2015; Sabir et al., 2020). Damaged plants can enhance antioxidant activity (SOD, POD, CAT, APX, and GR; **Figure 3**) and form antioxidant defence systems. In this study, ALA improved the stress tolerance in plants under Cd stress conditions. As the precursor of photosynthesis, ALA improved the activity level of heme molecules and eliminated excessive ROS (Ali et al., 2013b). Applying Si and Mn can also effectively reduce ROS levels, enhance antioxidant defence ability, and improve the resistance to harmful metals (Song et al., 2009; Farooq et al., 2013; Adrees et al., 2015). The discovery that Si can reduce MDA and ROS level in *S. miltiorrhiza* is consistent with previous findings in rice (Chen et al., 2019; Huang et al., 2021a), wheat (Huang et al., 2019), and cotton (Farooq et al., 2013). Application of ALA and Mn/Si alleviated Cd stress’s adverse effects by decreasing lipid peroxidation and increasing antioxidant enzyme activities (**Figures 2**, **3**).

In addition, the addition of ALA increased the resistance to Cd stress in *S. miltiorrhiza* roots and leaves. Compared with the control, Cd significantly reduced the chlorophyll concentration in *S. miltiorrhiza* leaves (**Table 2**) due to the influence on its metabolism level and the destruction of the photosynthesis mechanism, thus affecting plant growth. However, ALA significantly increased the chlorophyll concentration and photosynthetic gas exchange capacity of *S. miltiorrhiza*, and promoted plant growth (**Table 2**), which is consistent with previous findings (Youssef and Awad, 2008; Ali et al., 2013a). Adding Si can limit the transport of Cd from roots to leaves by accumulating a high concentration of Si in plant cell walls, and diluting the concentration of Cd by increasing biomass, thus alleviating the Cd stress in plants (Zhang et al., 2008a; Shi et al., 2013). Liang et al. (2005) showed that adding Si also increased soil pH and reduced the effectiveness of soil Cd, thus decreasing the Cd content in roots and leaves. Adding Mn improves the water loss, yellowing, and nutrient imbalance of leaves under Cd stress. Following the application of Mn in the soil, Mn hindered the migration of Cd to the root system. It reduced the absorption of Cd by *S. miltiorrhiza*, thus reducing the accumulation of Cd in plants (**Figure 4**). This is consistent with the findings of Huang et al. (2021b) and Wang et al. (2021b). In addition, Mn reduced the effectiveness of Cd in the soil and reduced the uptake and internal movement of Cd by plants.

From the perspective of gene regulation, Cd transporter proteins played a significant role in regulating cadmium uptake and transport. For example, in rice, boron and silicon downregulated the Cd transport genes, reduced the absorption and transport of Cd by plants, and reduced the accumulation of Cd in rice (Chen et al., 2019). The *HMA3* discussed in this paper is a cadmium transporter, and its primary function is to isolate cadmium from plant tissues to vacuoles. Previous studies have shown that overexpression of *HMA3* in tobacco plants can significantly improve the tolerance of plants to Cd (Cai et al., 2019). This is because vacuoles and vesicles of plants can contain a lot of toxic metals, and excessive Cd is transported into vacuoles or vesicles through transporter *HMA3*, thus reducing Cd toxicity (Miyadate et al., 2011; Yan et al., 2016). Plants can improve their resistance to adversity by chelating cytoplasmic heavy metal cations and transporting them to vacuoles of root cells. For *S. miltiorrhiza*, the use of ALA and Si/Mn both increased the expression of the *HMA3* gene under Cd pollution, and *HMA3* promoted a large amount of Cd to enter vacuole and be isolated, thus reducing the pollution of *S. miltiorrhiza*. In plants, Cd and Mn have a common transport system, like the *NRAMP* family proteins involved in the transport of Cd and Mn (Eruola, 2012; Ishimaru et al., 2012; Socha and Guerinot, 2014). In the experiment of Chang et al. (2020), knocking out the *NRAMP1* gene in rice reduced the Cd content in the plants, indicating that with the decrease of *NRAMP1* activity, the ability of plants to absorb Cd from the soil also decreased. *NRAMP1* gene is related to Cd absorption and transportation, mainly expressed in plant roots. Under Mn treatment, *NRAMP1* expression in roots was down-regulated, and Cd uptake by *S. miltiorrhiza* was reduced. In addition, in this study, when ALA and Si were co-treated with Cd-contaminated soil, the decrease in Cd content was more significant compared to ALA and Si application alone, which may be due to increased expression of *HMA3*, reduced Cd uptake from roots, and reduced roots transport to leaves. In contrast, the co-treatment of ALA and Mn increased the Cd content in plants, possibly due to the down-regulation of *HMA3* expression in leaves (**Figures 6**, **7**). In the present study, ALA/Si/Mn treatment increased the expression of ethylene and growth hormone-related genes (e.g., *ERF73*, *AMI1*, etc.) compared with Cd treatment alone (**Figures 6**, **7**), which helped to promote the growth and development of the plants, and the growth of *S. miltiorrhiza* plants was significantly improved under these treatments. Combinations of ALA and Mn/Si to Cd stressed plants activated ABA signalling pathway-related genes (such as *LEW3*), enhancing the adaptability of plants to adverse environments. In this study, ALA/Si/Mn treatment increased the expression of Ca^2+^ channel-related genes, which may induce the expression of plant essential enzyme genes and affect the synthesis of secondary metabolites of *S. miltiorrhiza*.

## Conclusion

The present findings showed that ALA could effectively promote the growth of Cd-stressed plants, and there was a good synergistic effect of Si/Mn. Under the joint treatment of ALA and Si/Mn, the antioxidant level was increased, ROS and MDA level was decreased, and the expression of genes related to stress resistance was changed. Moreover, ALA and Si jointly resisted Cd treatment and effectively reduced the Cd content in *S. miltiorrhiza*, which provides an effective and feasible method for reducing Cd accumulation in plants. Compared with ALA/Mn alone, the combined action of ALA and Mn promoted the accumulation of Cd, which provided a new idea for the remediation of Cd-contaminated soil. This experiment also shows that elements may interact with each other in some way, which affects the absorption and accumulation of heavy metals by plants and the resistance of plants to toxic heavy metals.

## Data availability statement

The original contributions presented in the study are included in the article/**Supplementary Material**. Further inquiries can be directed to the corresponding authors.

## Author contributions

YS: conceptualization, methodology, writing – original draft. XL: methodology, writing – original draft. UN: formal analysis, writing – review and editing. ZH: supervision. NB: supervision. ZY: supervision. BA: conceptualization, formal analysis, writing – review and editing. LX: conceptualization, formal analysis, writing – review and editing, funding acquisition.

## Funding

This work was supported by the National Natural Science Foundation of China (31871694), Fundamental Research Funds of Zhejiang Sci-Tech University (14042216-Y).

## Conflict of interest

The authors declare that the research was conducted in the absence of any commercial or financial relationships that could be construed as a potential conflict of interest.

The reviewer IK declared a past co-authorship with the author BA to the handling editor

## Publisher’s note

All claims expressed in this article are solely those of the authors and do not necessarily represent those of their affiliated organizations, or those of the publisher, the editors and the reviewers. Any product that may be evaluated in this article, or claim that may be made by its manufacturer, is not guaranteed or endorsed by the publisher.
