# Supplementary material for: Soil applied silicon and manganese combined with foliar application of 5-aminolevulinic acid mediate photosynthetic recovery in Cd-stressed Salvia miltiorrhiza by regulating Cd-transporter genes
Source: Front Plant Sci. 2022 Sep 29;13:1011872. doi: 10.3389/fpls.2022.1011872 (PMC9558727; doi:10.3389/fpls.2022.1011872)
Supplement: Supplementary file 1 [file DataSheet_1.doc]

**Table 1S.** The primers used for qRT-PCR.

| *Gene* | PCR primers |
| --- | --- |
| *ERF73* | F: 5′-CAGGCTGGATTGGGAATTTGCG-3′  R: 5′-CCATCAGCTCCTCCGACAG-3′ |
| *COL1* | F: 5′-CTCCCTCTTGACAACGGAGT-3′  R: 5′-AGAACTCCAACAGCCCTTCA-3′ |
| *XCT* | F: 5′-TCCGAGGTATTGGAGACTGC-3′  R: 5′-ACAGTCTTGGGTCTGCCTTT-3′ |
| *ETR1* | F: 5′-GCGTGCTCGTAACGATTTCT-3′  R: 5′-AGCCAACAGGTTACTGCTCT-3′ |
| *IAA4* | F: 5′-TGAATTACCCGGATCTGCTCA-3′  R: 5′-CATCTCCCATGGAACATCCC-3′ |
| *AXR3* | F: 5′-AAACGAGTCTGCTGGTTTGG-3′  R: 5′-CGACTCCTCTTCGCTCTTCT-3′ |
| *AMI1* | F: 5′-GCGAATGGGTTGAGTCAGTC-3′  R: 5′-GCATTGCTAGGATGCCGAAA-3′ |
| *PIN3* | F: 5′-CCAACTTCAATGTCGGCGAT-3′  R: 5′-CAGGGTAAGAAGCCATCGGA-3′ |
| *LEW3* | F: 5′-GGAATACATGGCTGCTGGTG-3′  R: 5′-ATCTCAAGCCGTTGTGATGC-3′ |
| *CDPK2* | F: 5′-GCCCGAGAATTTCCTCTTCG-3′  R: 5′-AATCACACCGGCACTCCATA-3′ |
| *RWA2* | F: 5′-CAGATGGTCAGCCGAAGATG-3′  R: 5′-CACCATAGCTGCAGCCAAAT-3′ |
| *MPK2* | F: 5′-TGCTCGTCTTTGACCCTTCA-3′  R: 5′-GGAGCAGCTTCTGGATGGTA-3′ |
| *MPK18* | F: 5′-GGCGTTGTAGGTTCTGCAAT-3′  R: 5′-CTCCGAGAAGGAGGAAGCAT-3′ |
| *MMK2* | F: 5′-CCAATCAACCTTTGGCCGAT-3′  R: 5′-GATGTTGTCCTTGCGAGACC-3′ |
| *MPK17* | F: 5′-CCCGAATGCTGATCCCTTTG-3′  R: 5′-TTTCGTGACAGGTTGTGCAG-3′ |
| *PTPA* | F: 5′-ACTACGTCGACGAAATCCCT-3′  R: 5′-CATTGCCAAAGCCATCCGTA-3′ |
| *CAX2* | F: 5′-GGTGAATCGAGTCAGCATCC-3′  R: 5′-GCGCTCTGCCAATGGAATAA-3′ |
| *ECA1* | F: 5′-CCTTTGTGGAGCCTCTGGTA-3′  R: 5′-GCTCTTTCGCTGGCAAACTA-3′ |
| *PML3* | F: 5′-TTCGTCGCCACCGTATAGAT-3′  R: 5′-CGGCCCAGATCCTTAATTGC-3′ |
| *ECA3* | F: 5′-CAACGGAAATCAGGGCAGAG-3′  R: 5′-GCATGCCAACCAACCCAATA-3′ |
| *MTP11* | F: 5′-TACTGGAGTCTGTTCGGCAA-3′  R: 5′-TCCGACAGAGTTGGTGATGA-3′ |
| *MTP8* | F: 5′-AGTTGGACAGTCAGCTCCTC-3′  R: 5′-CATGTGCCTCAATCAACGGT-3′ |
| *HMA3* | F: 5′-GGATCACAGAAGCAGCACAG-3′  R: 5′-GGAGCTCGAGCATTTCTTGG-3′ |
| *NRAMP1* | F: 5′-CGGAGCTACTAAGCTGGCTA-3′  R: 5′-AGTAAGGCGAAGCCACTTTC-3′ |
| *PAL* | F: 5′-TGTCGTCCACCTACCTCGTC-3′  R: 5′-CGCGGATCAAGTCCTTCTCG-3′ |
| *C4H* | F: 5′-ACCGATTCCGGTCCCAATCT-3′  R: 5′-AGGAGGAAGATGTCGCCGAA-3′ |
| *DXS2* | F: 5′-TCCGTGGTAGCAGCTCTTCA-3′  R: 5′-CAGTTCCTCGACCGAGAGGT-3′ |
| *DXR* | F: 5′-TGAGCCCGGTCGTATGACTT-3′  R: 5′-TGAACCAGCAGCAAGTGCAA-3′ |
| *HMGR3* | F: 5′-GGTGTTCAACAGCTCCAGCA-3′  R: 5′-TGCTGCACGAGAATCGGATG-3′ |
| *APX1* | F: 5′-GCTCCTATCATGCTCCGTCT-3′  R: 5′-CTTCCTGATGGGCTCCAAGA-3′ |
| *CSD2* | F: 5′-ACAAGGATCATGGCTCTCCC-3′  R: 5′-GTGAGCAACCCCTGCAATAAC-3′ |
| *FSD2* | F: 5′-TGGCTCGTGTATAAAGCAAATAGA-3′  R: 5′-CAGCAGCGGAGAGTAATCATAGA-3′ |
| *MSD1* | F: 5′-TGGAAGATGCCTGTTCGTGT-3′  R: 5′-GTCAAGCCTCACGTTCTTGT-3′ |
| *MSD2* | F: 5′-TTGCTCCTGTTCGTGTTGGT-3′  R: 5′-CAAGCCACACCCATCCTGAA-3′ |
| *PPT* | F: 5′-CTGGACAAGCCGATAGGGAC-3′  R: 5′-GCTATGGGCCTTGACCTTGT-3′ |
| *CPK6* | F: 5′-AGTCCCAGCAAAGAGAGCAT-3′  R: 5′-GGCGATCTCAGTGCACAAAT-3′ |
| *CPK4* | F: 5′-AGGGATTTGAAGCCCGAGAA-3′  R: 5′-ACTCCATACGTCTGCCTCAG-3′ |
| *ACA2* | F: 5′-AGTTACTGTCCGCATGGTCA-3′  R: 5′-GAAGAACGAGCCATCACCTG-3′ |
| *Actin* | F: 5′-GGTGCCCTGAGGTCCTGTT-3′  R: 5′-AGGAACCACCGATCCAGACA-3′ |

**Table 2S.** The classification and function of *Salvia miltiorrhiza* gene for qRT-PCR analysis.

| Gene category | *Gene* | Description |
| --- | --- | --- |
| ethylene-related genes | *ERF73* | Integrase-type DNA-binding superfamily protein |
| ethylene-related genes | *COL1* | CONSTANS-like 1 |
| ethylene-related genes | *XCT* | XAP5 family protein |
| ethylene-related genes | *ETR1* | Signal transduction histidine kinase, hybrid-type, ethylene sensor |
| auxin-related genes | *IAA4* | auxin-responsive protein IAA4 |
| auxin-related genes | *AXR3* | AUX/IAA transcriptional regulator family protein |
| auxin-related genes | *AMI1* | amidase 1 |
| auxin-related genes | *PIN3* | Auxin efflux carrier family protein |
| ABA signaling pathway genes | *LEW3* | UDP-Glycosyltransferase superfamily protein |
| ABA signaling pathway genes | *CDPK2* | calcium-dependent protein kinase 2 |
| ABA signaling pathway genes | *RWA2* | O-acetyltransferase family protein |
| MAPK cascade reaction genes | *MPK2* | mitogen-activated protein kinase homolog 2 |
| MAPK cascade reaction genes | *MPK18* | mitogen-activated protein kinase 18 |
| MAPK cascade reaction genes | *MMK2* | mitogen-activated protein kinase homolog MMK2 |
| MAPK cascade reaction genes | *MPK17* | MAP kinase 17 |
| MAPK cascade reaction genes | *PTPA* | protein phosphatase 2 phosphatase |
| Mn transport-related genes | *CAX2* | cation exchanger 2 |
| Mn transport-related genes | *ECA1* | ECA1 gametogenesis related family protein |
| Mn transport-related genes | *PML3* | PML nuclear body scaffold |
| Mn transport-related genes | *ECA3* | endoplasmic reticulum-type calcium-transporting ATPase 3 |
| Mn transport-related genes | *MTP11* | Cation efflux family protein |
| Mn transport-related genes | *MTP8* | Cation efflux family protein |
| Cd transport-related genes | *HMA3* | heavy metal atpase 3 |
| Cd transport-related genes | *NRAMP1* | natural resistance-associated macrophage protein 1 |
| *Salvia miltiorrhiza* component-related genes | *PAL* | PHE ammonia lyase |
| *Salvia miltiorrhiza* component-related genes | *C4H* | cinnamate-4-hydroxylase |
| *Salvia miltiorrhiza* component-related genes | *DXS2* | 1-deoxy-D-xylulose 5-phosphate synthase |
| *Salvia miltiorrhiza* component-related genes | *DXR* | 1-deoxy-D-xylulose 5-phosphate reductoisomerase |
| *Salvia miltiorrhiza* component-related genes | *HMGR3* | hydroxymethyglutaryl-CoA reductase |
| ROS scavenged genes | *APX1* | ascorbate peroxidase 1 |
| ROS scavenged genes | *CSD2* | copper/zinc superoxide dismutase 2 |
| ROS scavenged genes | *FSD2* | Fe superoxide dismutase 2 |
| ROS scavenged genes | *MSD1* | manganese superoxide dismutase 1 |
| ROS scavenged genes | *MSD2* | superoxide dismutase [Mn] |
| ROS scavenged genes | *PPT* | palmitoyl-protein thioesterase |
| calcium channel genes | *CPK6* | Calcium-dependent protein kinase family protein |
| calcium channel genes | *CPK4* | calcium-dependent protein kinase 4 |
| calcium channel genes | *ACA2* | calcium ATPase 2 |
| internal reference gene | *Actin* | a constant expression internal reference gene |
